# Clinical features and outcome of vertebral osteomyelitis after spinal injection: is it worth the price?

**DOI:** 10.1007/s15010-023-02024-9

**Published:** 2023-04-18

**Authors:** Ayla Yagdiran, Gregor Paul, Charlotte Meyer-Schwickerath, Justus Scheder-Bieschin, David Tobys, Nikolaus Kernich, Peer Eysel, Norma Jung

**Affiliations:** 1 Department of Orthopedics and Trauma Surgery, University Hospital of Cologne, Cologne, Germany; 2 Department of Internal Medicine III - Gastroenterology and Infectious Diseases, University Hospital of Augsburg, Stenglinstraße 2, 86156 Augsburg, Germany; 3 Division of Infectious Diseases, Department I of Internal Medicine, University of Cologne, Cologne, Germany; 4 Department of Interdisciplinary Acute, Emergency and Intensive Care Medicine (DIANI), Klinikum Stuttgart, Stuttgart, Germany; 5 Institute for Medical Microbiology, Immunology and Hygiene, Faculty of Medicine, University Hospital Cologne, University of Cologne, Cologne, Germany

**Keywords:** Vertebral osteomyelitis, Spinal injection, Psoas abscess, *Staphylococcus aureus*, Comorbidity, Survival

## Abstract

**Purpose:**

Spinal injections are increasingly used for back pain treatment. Vertebral osteomyelitis (VO) after spinal injection (SIVO) is rare, but patient characteristics and outcome have not been well characterized. The aim of this study was to assess patient characteristics of SIVO in comparison to patients with native vertebral osteomyelitis (NVO) and to determine predictors for 1-year survival.

**Methods:**

This is a single-center cohort study from a tertiary referral hospital. This is a retrospective analysis of Patients with VO who were prospectively enrolled into a spine registry from 2008 to 2019. Student’s *t*-test, Kruskal–Wallis test or Chi-square test were applied for group comparisons. Survival analysis was performed using a log-rank test and a multivariable Cox regression model.

**Results:**

283 VO patients were enrolled in the study, of whom 44 (15.5%) had SIVO and 239 (84.5%) NVO. Patients with SIVO were significantly younger, had a lower Charlson comorbidity index and a shorter hospital stay compared to NVO. They also showed a higher rate of psoas abscesses and spinal empyema (38.6% [SIVO] vs. 20.9% [NVO]). *Staphylococcus aureus* (27%) and coagulase-negative staphylococci (CNS) (25%) were equally often detected in SIVO while *S. aureus* was more frequently than CNS in NVO (38.1% vs. 7.9%).Patients with SIVO (*P* = 0.04) had a higher 1-year survival rate (Fig. 1). After multivariate analysis, ASA score was associated with a lower 1-year survival in VO.

**Conclusion:**

The results from this study emphasize unique clinical features of SIVO, which warrant that SIVO should be estimated as a separate entity of VO.

## Introduction

Spinal injections (SI) of glucocorticoids and/or analgesics are regularly used for short-term relief in patients with back pain due to radiculopathy or neurogenic claudication. However, its effectiveness is controversial [1–3]. Even though the procedure is generally considered safe, there can be far-reaching complications in rare cases. Some of these are hemorrhage, nerve injury, dural puncture, stroke, paralysis and death [4, 5]. These also include Infections, occurring in 0.01–0.1% of cases [5, 6]. Meningitis, epidural abscess and vertebral osteomyelitis (VO) are the most severe forms of infections. To date, two large series with nosocomial infections associated with spinal injections of contaminated steroids [7, 8] and a few case reports with SIVO [9, 10] have been reported.But whether the clinical presentation or outcome of SIVO differs from native vertebral osteomyelitis (NVO), which often results from hematogenous seeding, is entirely unknown. There are studies comparing NVO and postsurgical vertebral osteomyelitis (PVO), showing that there are microbiological differences between these groups. PVO is more likely to be caused by methicillin-resistant *Staphylococcus aureus*, coagulase-negative staphylococci and other skin flora bacteria [11, 12]. The relapse rate appears to be higher in PVO, but NVO patients have lower survival rates [11, 12]. For this reason, one assumes that it is rather a matter of different clinical entities of the same disease. To the best of our knowledge, no data are currently available on detailed clinical characteristics of SIVO. Hence, we analyzed data on patient characteristics, treatment modalities, outcomes and 1-year survival rate from patients with SIVO and compared these with patients with NVO.

## Materials and methods

### Patients

In this single-center cohort study, we retrospectively analyzed all patients from a tertiary referral hospital with confirmed VO who were prospectively enrolled from January 2008 to December 2019 in the European “Spine Tango” and since 2016 the German “Deutsche Wirbelsäulengesellschaft (DWG)” registry. Inclusion criteria were presence of characteristic back and/or leg pain plus characteristic magnetic resonance imaging (MRI) or an abscess or vertebral body destruction detected by computed tomography (CT).

All cases were discussed in an interdisciplinary approach between orthopedic surgeons and infectious disease specialists to confirm or rule out the diagnosis of VO. The relevance and plausibility of the identified microorganism from blood cultures or intraoperative specimens obtained by surgery or CT-guided puncture were checked by an infectious disease specialist separately. Detection of a virulent organism such as *Staphylococcus aureus* or gram-negative bacteria in one specimen or the detection of a low-virulence organism such as coagulase-negative staphylococci from the normal skin flora in at least two relevant samples were considered to be the etiologic microorganism.

### Data collection and definitions

The following data were collected after enrollment: age, gender, length of hospital stay, affected spinal segment, American Society of Anesthesiologists Physical Status Classification System (= ASA). The ASA score was developed in 1941 to classify patient comorbidities and is widely used by clinicians [13]. It ranges from ASA 1—A normal healthy patient, to ASA 5—A moribund patients who is not expected to survive without surgery.

In addition, the following demographic and clinical parameters were recorded for all VO patients: presence of bacteremia, causative pathogens, body mass index (BMI), laboratory tests, comorbidities (diabetes, oncologic disease, autoimmune disease, chronic obstructive pulmonary disease (COPD), inflammatory bowel disease (IBD), rheumatic disease, chronic heart failure, chronic renal failure, infectious endocarditis (IE), osteoporosis, alcohol and drug abuse), Charlson comorbidity index (= CCI), clinical manifestations of VO such as presence of psoas abscess, empyema and pre-operative neurological deficit.

Bone destruction of the vertebral bodies was noted according to the Eysel/Peters classification for spondylodiscitis [14]. It is defined as follows:

- Stage 0: No destruction
- Stage 1: Reduction of the intervertebral space
- Stage 2: Erosion of the base- and upper plates of the vertebral bodies
- Stage 3: Spinal deformity with kyphosis
- Stage 4: Reactive bone formation and kyphotic malalignment

The Frankel scale categorizes the extent of functional or neurological impairment [15]:

- Grade A: No motor or sensory function below the lesion level
- Grade B: No motor function, but some sensation is retained below the lesion level
- Grade C: Some motor functions with no practical application
- Grade D: Useful motor function below the lesion level
- Grade E: Normal motor and/or sensory function, may have reflex abnormalities.

Relapse was defined as recurrent symptoms after stopping the first course of antibiotic treatment and receiving a second course for the initial pathogen responsible for VO. After enrollment in the Spine Tango or later DWG registry, patients were regularly invited for follow-up visits or phone interviews at defined time points (3, 6, 9, 12 months).

### Ethics

The study was approved in the year 2009 by the local ethics committee (vote 09-182). All patients gave their informed consent to be registered within Spine Tango or DWG.

### Statistical analysis

Continuous data were expressed as mean with standard deviation (SD) in the case of normal distribution or as median with interquartile range (IQR) for non-normally distributed data. Categorical variables were presented as number (*n*) and percentage (%). Statistical differences between groups were determined using Student’s *t*-test (continuous variables with normal distribution), Kruskal–Wallis test (continuous variables not normally distributed), or Chi-square test and Fisher’s exact test (categorical variables). In case of multiple comparison, a Bonferroni correction was used. A Shapiro–Wilk test was performed to test for normality. Survival analysis was carried out using a log-rank test (Mantel–Cox method). Cox regression analysis was performed to analyze the influence of clinically relevant covariates on survival. These variates were included in the multivariate regression model using the backward, stepwise selection procedure. Reported p-values are two-tailed, with *P* < 0.05 considered statistically significant. SPSS (SPSS 24, SPSS Inc., Armonk NY, USA) was used for statistical analysis.

## Results

### Patient characteristics and disease manifestation

**Fig. 1:**
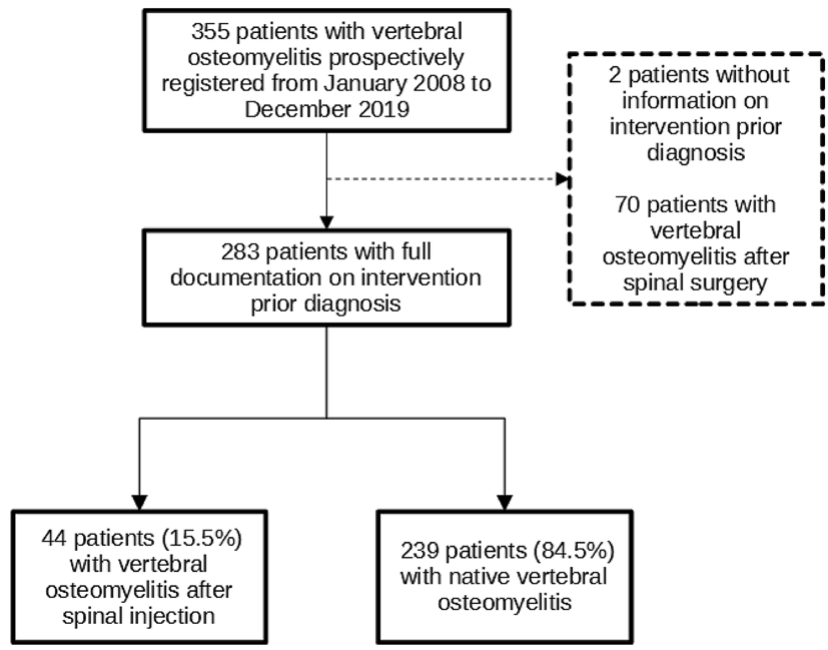
Flow diagram showing study population selection

The study population selection is depicted in Fig. 1.

A total of 283 patients were enrolled in this study, of whom 44 (15.5%) had SIVO and 239 (84.5%) had native vertebral osteomyelitis (NVO). Demographics and comorbidities are listed in Table 1. Male patients were predominantly affected, but the gender distribution was the same in both groups (*P* = 0.73). The median age of SIVO patients was 66.5 years, which is significantly younger than NVO patients (70.5 years; *P* = 0.02). SIVO patients had less comorbidities (mean 0.95 vs. 1.38, *P* = 0.03) than NVO, which is reflected in a lower Charlson Comorbidity Index (CCI) (*P* = 0.006). Specifically, they had less often chronic renal failure (6.8% vs. 18.8%, *P* = 0.04) and chronic heart failure (6.8% vs. 21.3%, *P* = 0.02).

**Table 1:** Demographics and comorbidities of patients with native vertebral osteomyelitis and vertebral osteomyelitis after spinal injection

	Total *N* = 283			Native *N* = 239			Spinal injection *N* = 44			*P* value
	*N*	(%)	*N*_missing_	*N*	(%)	*N*_missing_	*N*	(%)	*N*_missing_	
Female	98	(34.6)	0	84	(35.1)	0	14	(31.8)	0	0.73
Age, years, median (IQR)	70	(60.5–77)	0	70.5	(61–78)	0	66.5	(57–71)	0	0.02
BMI, kg/m^2^, median (IQR)	25.7	(22.4–29.45)	30	25.6	(22–29.4)	23	26.8	(23.25–31.35)	7	0.13
Number of comorbidities, mean (SD)	1.3	(1.18)	0	1.38	(1.15)	0	0.95	(1.3)	0	0.03
Charlson comorbidity index			0			0			0	
CCI 0	11	(3.9)		8	(3.4)		3	(6.8)		0.006
CCI 1–2	66	(23.3)		50	(20.9)		16	(36.4)		
CCI 3–4	93	(32.8)		77	(32.2)		16	(36.4)		
CCI 5 and above	113	(39.9)		104	(43.5)		9	(20.5)		
Conditions										
Autoimmune disease	18	(6.4)	0	14	(5.9)	0	4	(9.1)	0	0.5
Diabetes	72	(25.4)	0	63	(26.4)	0	9	(20.5)	0	0.46
Alcohol Abuse	19	(6.7)	0	15	(6.3)	0	4	(9.1)	0	0.51
Intravenous drug use	12	(4.2)	0	11	(4.6)	0	1	(2.3)	0	0.7
Malignancy	66	(13.3)	0	55	(23)	0	11	(25)	0	0.85
Chronic renal failure	48	(16.9)	0	45	(18.8)	0	3	(6.8)	0	0.04
Chronic heart failure	54	(19.1)	0	51	(21.3)	0	3	(6.8)	0	0.02
Endocarditis	16	(5.7)	0	16	(6.7)	0	0	(0)	0	0.15

*IQR* interquartile range, *SD* standard deviation, *CCI* Charlson comorbidity index, *BMI* body mass index

The disease presentation also differed between the groups, as shown in Table 2. Patients with SIVO had more often psoas abscesses (38.6% vs. 20.9%, *P* = 0.02) and spinal empyemas (45.5% vs. 30.1%, *P* = 0.05). The spinal level, the number of segments involved, the degree of neurological deficit measured by the Frankel scale and the extent of bone destruction as measured with the Eysel/Peters classification for bone destruction in VO did not differ.

**Table 2:** Disease presentation, microbiological etiology, and laboratory analysis

	Total *N* = 283			Native *N* = 239			Spinal injection *N* = 44			*P* value
	*N*	(%)	*N*_missing_	*N*	(%)	*N*_missing_	*N*	(%)	*N*_missing_	
Level, %			0			0			0	
Cervical	11	(3.9)		10	(4.2)		1	(2.3)		0.1
Thoracic	91	(32.2)		82	(34.3)		9	(20.5)		
Lumbar	165	(58.3)		132	(55.2)		33	(75)		
Multifocal	16	(5.7)		15	(6.3)		1	(2.3)		
Segments involved, mean (SD)	1.3	(0.6)	0	1.3	(0.7)	0	1.2	(0.5)	0	0.46
Spinal empyema	92	(32.5)	0	72	(30.1)	0	20	(45.5)	0	0.05
Psoas abscess	67	(23.7)	0	50	(20.9)	0	17	(38.6)	0	0.02
Destruction			0			0			0	
0	6	(2.5)		6	(2.5)		0	(0)		0.13
1	20	(7.1)		15	(6.3)		5	(11.4)		
2	144	(50.9)		117	(49)		27	(61.4)		
3	113	(39.9)		101	(42.3)		12	(27.3)		
4	0	(0)		0	(0)		0	(0)		
Frankel scale			0			0			0	
A	5	(1.8)		5	(2.1)		0	(0)		0.18
B	17	(5.3)		13	(5.4)		2	(4.5)		
C	28	(7.1)		17	(7.1)		3	(6.8)		
D	33	(7.4)		14	(5.9)		7	(15.9)		
E	270	(78.4)		190	(79.5)		32	(72.7)		
Bacteremia	116	(41.3)	2	99	(41.8)	2	17	(38.6)	0	0.74
Microbe			0			0			0	0.04
* Staphylococcus aureus*	103	(36.4)		91	(38.1)		12	(27.3)		< 0.001
MRSA	11	(10.7)		10	(10.9)		1	(8.3)		
CNS	30	(10.6)		19	(7.9)		11	(25)		< 0.001
* Enterococcus* spp. and *Streptococcus* spp.	34	(12)		29	(12.1)		5	(11.4)		0.89
Gram-negative bacteria	27	(9.5)		24	(10)		3	(6.8)		0.5
Other*	19	(6.7)		16	(6.7)		3	(6.8)		1
Laboratory analysis										
Hemoglobin, g/dl (median, IQR)	10.7	(9.7–12.1)	0	10.6	(9.5–11.9)	0	11.6	(10.3–13.2)	0	0.001
Thrombocytes, T/µl (median, IQR)	312	(239.5–408)	0	317	(249–418)	0	273.5	(221–369.75)	0	0.13
Leukocytes, T/µl (median, IQR)	9.1	(7–11.9)	0	8.9	(6.9–11.8)	0	10.4	(7.4–12.1)	0	0.37
Albumin, mg/dl (median, IQR)	32	(27–36)	1	31	(27–36)	1	34.5	(29–38)	0	0.02
C-reactive protein, mg/l (median, IQR)	72.1	(31.3–139.9)	8	69.7	(32.4–132.8)	6	79.1	(27.5–151.6)	2	0.63
Glomerular filtration rate, ml/min (median, IQR)	75	(50–98)	9	73	(49–98)	8	83	(61.5–99.5)	1	0.26
Quick, % (mean, IQR)	93	(76–106)	10	92	(76–104.5)	8	100	(78–108)	2	0.13

A Bonferroni correction was used for multiple comparison in case of microbiological etiology. A *P* value below 0.0028 was considered statistically significant

*IQR* interquartile range, *SD* standard deviation, *CNS* Coagulase-negative staphylococci

*In detail: SIVO – *N* = 1 *Parvimonas micra*, *N* = 1 *Peptococcus* spp., *N* = 1 *Aspergillus fumigatus* NVO – *N* = 3 *Mycobacterium tuberculosis*, *N* = 2 *Mycobacterium bovis*, *N* = 1 *Mycobacterium xenopi*, *N* = 3 *Parvimonas micra*, *N* = 1 *Cutibacterium acnes*, *N* = 1 *Bacteroides fragilis*, *N* = 1 *Eikenella corrodens*, *N* = 1 *Peptoniphilus tyrrelliae*, *N* = 2 *Candida tropicalis*, *N* = 1 *Candida albicans*

The type of microbe causing VO was significantly different between the groups (*P* = 0.04). The most frequently detected bacteria in both groups were *Staphylococcus aureus*, coagulase-negative staphylococci (CNS), *Enterococcus* spp. and *Streptococcus* spp. Gram-negative bacteria were the least common. The percentage of *S. aureus* infections (27.3%) and CNS infections (25%) were nearly equal in SIVO, while infections by *S. aureus* were more common in comparison to CNS in NVO (38.1% vs. 7.9%). Empirical therapy for VO in our center was Flucloxacillin and Ceftriaxone. In patients with detectable microorganisms, available susceptibility testing and breakpoints according to European Committee on Antimicrobial Susceptibility Testing (EUCAST), the empirical regimen was considered effective in 110 out of 165 patients in the NVO group (66.6%) and 19 out of 32 patients in the SIVO group (56%) (*P* = 0.43).

### Therapy, outcome and survival

Data on therapeutic modalities and outcome are summarized in Table 3. Patients with SIVO had a significantly shorter hospital stay (mean 29.5 vs 35.9 days, *P* = 0.04) and a lower ASA score (*P* = 0.002). The rate of surgical intervention (*P* = 0.32), type of surgery (*P* = 0.83) and surgical technique (*P* = 0.18) did not differ between groups.

**Table 3:** Therapy and outcome

	Total *N* = 283			Native *N* = 239			Spinal Injection *N* = 44			*P* value
	*N*	(%)	*N*_missing_	*N*	(%)	*N*_missing_	*N*	(%)	*N*_missing_	
Length of hospital stay, days (mean, SD)	34.9	(21.9)	0	35.9	(22.7)	0	29.5	(16.3)	0	0.04
ASA			14			9			5	
1	9	(3.2)		6	(2.5)		3	(6.8)		0.002
2	70	(24.7)		53	(22.2)		17	(38.6)		
3	156	(55.1)		140	(58.6)		16	(36.4)		
4	34	(12)		31	(13)		3	(6)		
5	0	(0)		0	(0)		0	(0)		
Therapy			0			0			0	
Conservative	33	(11.7)		26	(10.9)		7	(15.9)		0.32
Surgical	250	(88.3)		213	(89.1)		37	(84.1)		
Type of surgery			0			0			0	
1-stage	119	(42)		102	(42.7)		17	(38.6)		0.83
2-stage	131	(46.3)		111	(46.4)		20	(45.5)		
OP technique			0			0			0	
Bone graft	85	(30)		75	(13.3)		10	(22.7)		0.18
Cage	112	(39.6)		88	(36.8)		24	(54.5)		
Combination	8	(2.8)		6	(25.1)		2	(4.5)		
Relapse, %	23	(8.1)	0	13	(5.4)	0	4	(9.1)	0	0.31

*SD* standard deviation, *ASA* American Society of Anesthesiologists physical status classification system

**Fig. 2:**
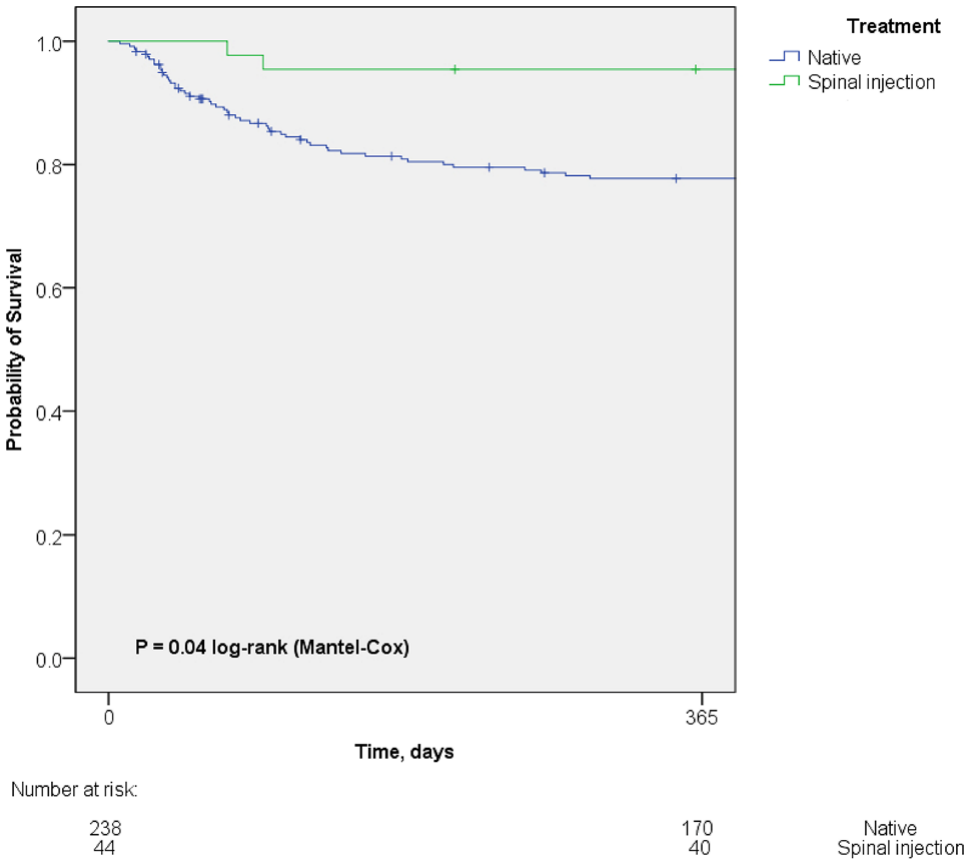
Kaplan–Meier survival curve of patients with vertebral osteomyelitis after spinal injection compared to native vertebral osteomyelitis. Kaplan–Meier analysis depicts a better 1-year survival rate of patients with vertebral osteomyelitis after spinal injection in comparison to vertebral osteomyelitis after spinal injection (log-rank *P* = 0.04). No survival data were available for one patient in the NVO group. In the NVO group 17 patients (7%) were lost to follow-up and 2 patients (4.5%) in the SIVO group

A log-rank test was performed to determine if there were differences in the 1-year survival for the different types of VO. One-year survival of patients with SIVO was significantly better compared to patients with NVO (*P* = 0.04). The Kaplan–Meier curve is shown in Fig. 2. Of the patients that died within 1 year after diagnosis of VO, 18 out of 51 patients (35%) had *S. aureus* bacteremia in the NVO group and none out of two in the SIVO group.

A backward stepwise cox regression was used to explore the influence of potential predictors on 1-year mortality in VO (Table 4). Starting with the variables age, NVO vs. SIVO, ASA score and *Staphylococcus aureus* that might theoretically be good predictors based on clinical relevance, only ASA score (HR 1.82, 95% CI 1.18–2.8, *P* = 0.007) has shown to be a predictor of 1-year survival in VO.

**Table 4:** Multivariable Cox regression for mortality risk after 1 year

	Hazard ratio	95% CI for Hazard ratio		*P* value
		Lower	Upper	
ASA Score	1.82	1.18	2.8	0.007

A backward stepwise cox regression was used to explore the influence of potential predictors on 1-year mortality in vertebral osteomyelitis out of the following candidate variables: age, native (NVO) vs. vertebral osteomyelitis after spinal injection (SIVO), ASA score and *Staphylococcus aureus*

*NVO* native vertebral osteomyelitis, *SIVO* vertebral osteomyelitis after spinal injection, *ASA* American Society of Anesthesiologists, *CI* confidence interval

## Discussion

We present a comprehensive analysis of clinical features and 1-year survival of SIVO patients in comparison to the reference VO population NVO. In detail, the data (i) suggest that patients with SIVO are younger and have fewer comorbidities. (ii) The low virulence microorganisms CNS more frequently caused SIVO than NVO which could be expected. (iii) Survival analysis indicates that mortality is lower in SIVO and that the ASA score is the only significant predictor of survival in patients with VO.

The reason for the younger age in patients with SIVO is unclear, but studies show that the elderly are more often affected by NVO [16, 17]. Since age is significantly associated with comorbidities and mortality in VO, the lower CCI and better survival in patients with SIVO is in part explained by the age difference [18]. Also, the higher number of infections caused by *S. aureus* may contribute to the lower survival in NVO as shown in other studies and conversely to the better survival in SIVO [17]. Numerically, we found a higher number of patients with *S. aureus* bacteremia in deceased patients in the NVO group (35%) compared to the SIVO group (0%). Since only two patients died in the SIVO group, no statistical subgroup analysis was possible and the data should be viewed with caution.

Most guidelines, for instance from the USA, Great Britain or Germany, do not recognize SIVO as a separate disease entity [19–21]. Nevertheless, the difference in the causative microorganisms could require separate empiric anti-infective regimens to mitigate the divergence in survival. As other studies have shown, the prevalence of CNS is higher after spinal interventions such as SI or surgery [22]. In Germany, beta-lactam antibiotics are often used as empiric therapy for VO, since the rate of methicillin-resistant *S. aureus* (MRSA) is less than 10% [23]. Due to the high rate of resistance of CoNS to oxacillin and other beta-lactams, as a result such empiric therapy would likely lead to higher treatment failure rates in SIVO [24, 25].

To the best of our knowledge, our study is the first to assess SIVO in detail and compare this VO group to other VO group to outline the clinical differences. Whereas the clinical features of PVO have been studied before and show some similarities to SIVO. Similar to SIVO, patients with PVO have fewer comorbidities, a lower ASA score and VO was less often caused by *S. aureus*, but more often by CNS [11, 12]. Also, survival in PVO is better in comparison to the reference VO population NVO, which is in line with the survival of SIVO compared to NVO. The higher rate of infections with CNS in PVO and SIVO is most likely explained by the breach of the skin barrier, which leads to the introduction of commensal skin bacteria in both VO groups. But there are notable differences between SIVO and PVO as well. Both Breuninger et al. and Kim et al. show that the rate of bacteremia is higher in PVO than in NVO [11, 12]. Whereas in our study, no difference was found regarding the rate of bacteremia between SIVO and NVO. The reason and pathophysiology for this phenomenon is unclear. Furthermore, SIVO shows a higher rate of epidural empyemas/abscesses, which is also true for PVO. Likewise, we show a higher rate of psoas abscesses. Psoas abscess and intraspinal empyema are interconnected disease manifestations and often result from a local contiguous source. Psoas abscess formation after SI has mostly been reported in individual case reports and has been considered to be rare [26, 27]. Nevertheless, in our cohort, more than a third of patients with SIVO developed psoas abscesses. The reason for the higher rate of psoas abscesses compared to the other disease manifestations of VO remains elusive.

Treatment algorithms for spinal infections are often controversial, as therapy is highly individualized. In the presence of intraspinal empyema, surgery is more often recommended than in VO patients without intraspinal empyema [28]. In our cohort, the rate of surgical therapy was nearly equal in SIVO and NVO, although spinal empyema was more common in SIVO. However, the long-term result was better in SIVO. This challenges the concept of urgent surgery in most patients with intraspinal empyema and warrants further investigation into which patient population with intraspinal empyema may benefit from surgery with regard to their outcome.

One main problem is clearly the precise definition of SIVO. It is a chicken-and-egg problem. SI can obviously cause VO, and as lower back pain is a common problem in the general population and SI numbers are increasing, we will consequently see more cases of SIVO in the future [1, 29, 30]. On the other hand, it is known that the diagnosis of VO is often delayed up to several months because of the nonspecific clinical presentation and many patients receive SI because of the assumption of the presence of nonspecific back pain [31, 32]. This is clearly a limitation, but the distinctive clinical features between SIVO and NVO, with a high rate of CNS causing infections in SIVO, suggest that case selection was appropriate for most patients in our study. Ideally, an MRI scan should precede the first SI session to rule out spinal infections, especially in patients with clinical signs of infection (e.g., fever, chills) or elevated inflammatory markers. Though, the study by Cohen et al. has shown that an MRI prior SI does not improve outcome and has little impact on clinical decision making [33]. Since the study did not pay attention to the possibility of spinal infection prior to SI, this statement cannot be generalized for SI. We do believe that an MRI prior to SI is not only necessary but reasonable with regard to the rising incidence of VO to minimize the risk of SIVO. More limitations of our study are the single-center approach and setting in a tertiary center, which is likely to introduce a selection bias, as more severe cases are referred to our center. Although we present the largest cohort of patients with SIVO compared to other VO groups that have been systematically studied, studies with larger cohorts would be desirable to gain more statistical power.

## Conclusions

In summary, we show for the first time that SIVO has differing clinical features in comparison to the reference VO population NVO. Like PVO, SIVO has a higher rate of CNS, patients are younger and have fewer comorbidities than patients with NVO. SIVO patients have a high rate of psoas abscesses. Although, survival is better in patients with SIVO, the associated morbidity prompts a reevaluation of SI of glucocorticoids given the unclear efficacy on the short- and long- term effect on back pain.

## Data Availability

Raw data are not publicly available to preserve individuals’ privacy under the European General Data Protection Regulation. Anonymized data are available upon reasonable request.
